# Morphological Features of Organelles during Apoptosis: An Overview 

**DOI:** 10.3390/cells2020294

**Published:** 2013-05-07

**Authors:** Maria Grazia Bottone, Giada Santin, Francesca Aredia, Graziella Bernocchi, Carlo Pellicciari, Anna Ivana Scovassi

**Affiliations:** 1 Laboratorio di Biologia Cellulare e Neurobiologia, Dipartimento di Biologia e Biotecnologie “L. Spallanzani”, Università di Pavia, 27100 Pavia, Italy; E-Mails: bottone@unipv.it (M.G.B.); giadasantin@gmail.com (G.S.); bernocchi@unipv.it (G.B.); pelli@unipv.it (C.P.); 2 Istituto di Genetica Molecolare CNR, 27100 Pavia, Italy; E-Mail: aredia@igm.cnr.it

**Keywords:** apoptosis, cytoskeleton, endoplasmic reticulum, Golgi apparatus, lysosomes, mitochondria

## Abstract

An apoptotic program leading to controlled cell dismantling implies perturbations of nuclear dynamics, as well as changes affecting the organelle structure and distribution. In human cancer cells driven to apoptosis by different stimuli, we have recently investigated the morphological properties of several organelles, including mitochondria, lysosomes, endoplasmic reticulum and Golgi apparatus. In this review, we will discuss the body of evidence in the literature suggesting that organelles are generally relocated and/or degraded during apoptosis, irrespectively of the apoptogenic stimulus and cell type.

## 1. Introduction

The apoptotic program of controlled cell dismantling is characterized by a stereotypic series of cellular events that affect the inner cell structures, both the nucleus and cytoplasm, undergoing heavy restructuring. The most characteristic nuclear changes involve chromatin while other crucial events occur in the cytoplasmic compartment and alter organelle structure and function. 

In fact, the reorganization of the vacuolar system (*i.e.*, endoplasmic reticulum, Golgi apparatus and lysosomes) as well as of microfilaments and microtubules has been documented in apoptotic cells. The perturbation of organelle location and dynamics suggests that a program exists to manage cell demise, involving the whole cellular organization and governed by soluble factors, kinases and enzymes [1,2].

Evidence in the literature demonstrates that organelle reorganization during apoptosis is a largely stereotypic process taking place irrespective of the inducing apoptogenic stimulus [3,4,5]: this is the reason why, in the present review, only a few examples are given in the original figures presented.

## 2. Cytoskeleton

It is widely known that apoptotic cells reorganize their cytoskeleton, with fragmentation of the microfilament bundles: this confers plasticity to the whole cell and promotes volume decrease as well as cell shrinkage [6]. In fact, the immunocytochemical detection of actin and tubulin revealed that apoptogenic stimuli affect their organization, leading to the conversion of cytoskeletal threads into a cortical ring. Tubulin cytoskeleton reorganizes into thick bundles; actin microfilaments also form bundles that become progressively thicker, especially at the cell periphery [7].

**Figure 1:**
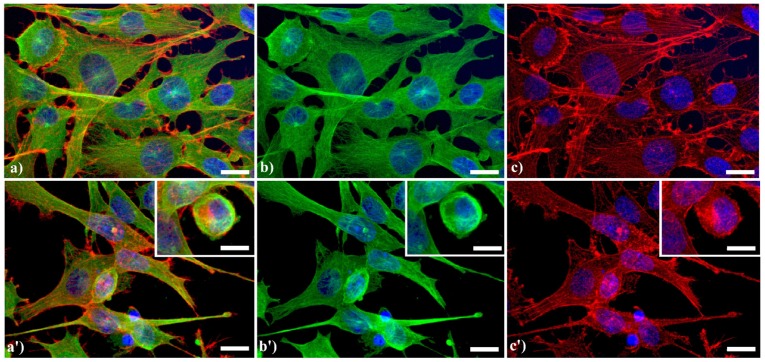
Fluorescence microscopy after double immunocytochemical detection of cytoskeletal components in B50 neuroblastoma cells. (**a**–**c**) control cells; (**a'**–**c'**) cells treated with 40 μM cysPt for 48 h. Cells on coverslips were fixed and processed for microfilaments (actin), labeled with Alexa 594-conjugated phalloidin (1:40 in phosphate buffered saline (PBS), red fluorescence; Molecular Probes, Invitrogen); microtubules were labeled with monoclonal anti-tubulin antibody (1:200 in PBS) revealed with Alexa 488-conjugated anti-mouse antibody (1:200 in PBS, green fluorescence; Molecular Probes). Cells were counterstained for DNA with 0.1 μg/mL Hoechst 33258 (blue fluorescence), washed with PBS and then mounted in Mowiol (Calbiochem) for microscopy analysis. Scale bar: 20 μm. Inset represents a late apoptotic cell.

A typical example of cytoskeleton changes during apoptosis is shown in Figure 1. The treatment of human B50 neuroblastoma cells with cisplatin (cisPt) affects the canonical regular distribution of microtubules visible as green fluorescence in control cells (a–c), promoting their aggregation in thick bundles after drug injury (a'–c'); actin filaments (red fluorescence), generally distributed at the cell periphery (a–c), underwent a crumbling in apoptotic cells (a'–c'). 

## 3. Endoplasmic Reticulum

**Figure 2:**
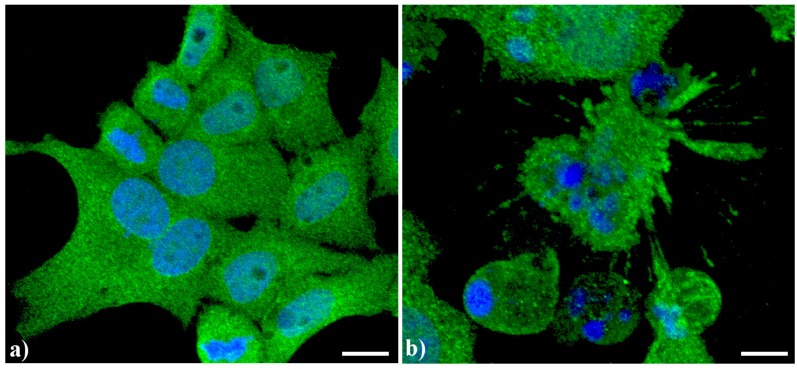
Confocal optical sections after immunolabeling for Endoplasmic reticulum (ER). (**a**) Control HeLa cells; (**b**) Cells treated with 1 μg/mL actinomycin D for 20 h. Cells were fixed and incubated with monoclonal anti-ER marker antibody (1:100 in PBS) (Abcam, Cambridge), revealed with Alexa 488-conjugated anti-mouse antibody (1:200 in PBS, green fluorescence; Molecular Probes). DNA was counterstained with Hoechst 33258 (blue fluorescence), washed with PBS and then mounted in Mowiol for confocal microscopy analysis. Scale bar: 20 μm.

Endoplasmic reticulum (ER) has a peculiar structure made by tubules dynamically moving and fusing with each other. Their membranes could be physically associated to mitochondria thus forming a complex modulated by a number of proteins and possibly involved in death signal propagation [8]. As previously shown [5,7], in apoptotic cells, prominent changes are observed in the organization of the vacuolar system, with ER greatly enlarged and swollen compared to the controls. In control human tumor HeLa cells (Figure 2a), ER results homogeneously spread in the whole cytoplasm with a major density in the perinuclear area. In actinomycin D-treated cells under conditions leading to apoptosis [9] (Figure 2b), the cisternae appeared more thickened with a less uniform distribution in the cytoplasm as well as the appearance of some dense vesicles.

## 4. Golgi Apparatus

This organelle is primarily involved in packaging proteins, driving them to different subcellular compartments and promoting secretion, and works in cooperation with other organelles. Golgi apparatus (GA) is very important to face the noxious effects of oxidative stress, being considered a “stress sensor” [10]. As summarized by Jiang *et al.* [10], under apoptotic conditions the GA undergoes morphological and functional changes, including fragmentation, swelling and distension.

We have observed [7,11] that in control HeLa cells the GA exhibits its typical “ribbon like” shape, with flattened cisternae mainly located in the perinuclear area, whereas the vesicles immunopositive for the Golgi markers are progressively less numerous, toward the cell periphery. In cells treated with an apoptogenic stimulus, the GA undergoes fragmentation and redistributes in the cytoplasm, sometimes forming dense aggregates [7,11,12]. 

**Figure 3:**
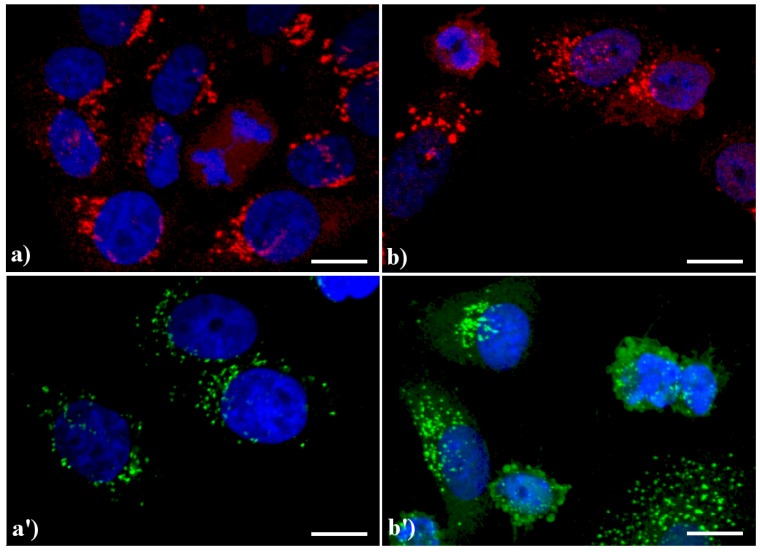
Confocal optical sections after immunolabeling for Golgi apparatus (GA). (**a**, **a'**) control HeLa cells; **(b)** cells treated with 1 μg/ml actinomycin D for 20 h; (**b'**): cells treated with 40 μM etoposide for 20 h. Cells on coverslips were fixed and incubated with monoclonal anti-golgin antibody (1:200 in PBS), revealed either with Alexa 594-conjugated anti-mouse antibody (1:200 in PBS, red fluorescence; Molecular Probes (**a**, **b**) or with Alexa 488-conjugated anti-mouse antibody (1:200 in PBS, green fluorescence; Molecular Probes (**a'**, **b'**). DNA was counterstained with Hoechst 33258 (blue fluorescence). Scale bar: 20 μm.

A similar reorganization of this organelle is shown in Figure 3: in control HeLa cells (a, a') the GA displays the typical semilunar shape in the perinuclear region; after apoptogenic treatment with actinomycin D (b) or etoposide (b'), the cisternae appear more scattered throughout the cytoplasm, and in late apoptosis a few Golgi-derived vesicles are visible.

## 5. Lysosomes

Lysosomes play a fundamental role in the intracellular degradation of endocytosed macromolecules and in regulating the correct turnover of long-lived proteins and organelles. They are involved in multiple pathways of cell death, including apoptosis, necrosis and autophagy [13,14,15]. In the apoptotic process, they are not concerned directly as entire organelles, but their enzymatic content (proteases such as cathepsins) plays a crucial degradative function being in charge for protein degradation [16]. the key activation step for lysosome function in apoptosis is represented by the destabilization of lysosomal membranes (also referred as lysosomal membrane permeabilization, LMP) to allow the release of lysosomal content into the cytosol [17]. Then, proteases cleave apoptotic players, such as Bcl-2 family members, possibly regulating also mitochondrial function [18,19].

**Figure 4:**
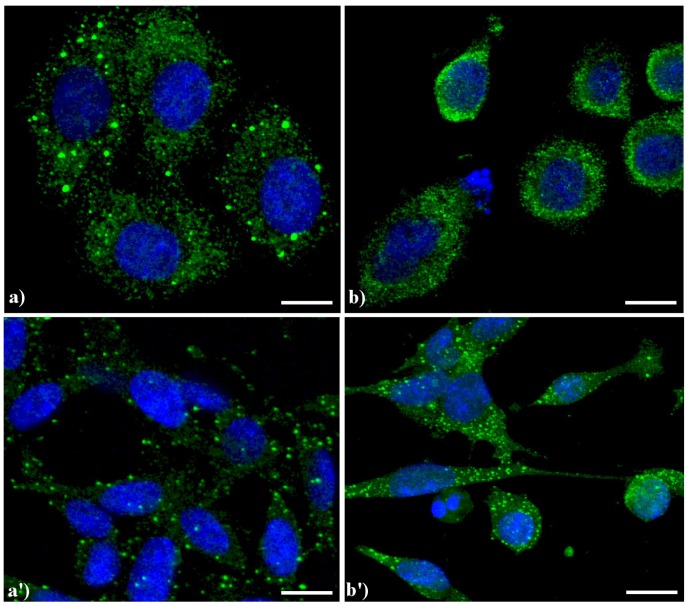
Confocal microscopy of immunolabeling of lysosomes. (**a**) control HeLa cells; (**b**) HeLa cells treated with etoposide (40 μM for 20 h); (**a'**) B50 control cells; (**b'**) B50 cells treated with cysPt (40 μM for 48 h). Cells were fixed and incubated with a human autoimmune serum (1:200 in PBS) recognizing lysosomal proteins (a kind gift of Dr. Claudia Alpini, IRCCS San Matteo, Pavia, Italy [20]) revealed with Alexa 488-conjugated anti-human IgG (1:200 in PBS, green fluorescence; Molecular Probes). DNA was counterstained with Hoechst 33258 (blue fluorescence). Scale bar: 20 μm.

Compared to the controls (Figure 4a), the lysosomes were apparently more numerous and smaller in HeLa cells treated with etoposide (b), and similar results were obtained in B50 cells treated with cisPt (a', b'). 

## 6. Mitochondria

Mitochondria are pivotal in the control of apoptosis, being involved not only in the intrinsic but also in the extrinsic pathway. After the pioneering works by Kroemer and his coworkers, who formulated the hypothesis that “permeabilization of mitochondrial membranes would be a decisive step in apoptosis” [21], many events leading to changes in the density and intracellular localization of mitochondria as well as to alterations in mitochondrial membrane potential have been described [22]. 

To monitor mitochondria pattern and distribution, it is possible to follow the localization of specific mitochondrial proteins, such as the mitochondrial isoform of the heat shock protein 70 (mtHSP70). This strategy allowed us to visualize rearrangements of the organelles in cells driven to apoptosis by different stimuli [23,24]. In untreated cells, mitochondria present their typical cytoplasmic distribution while in apoptotic cells they show a reduction of about 30% and form dense perinuclear masses. 

**Figure 5:**
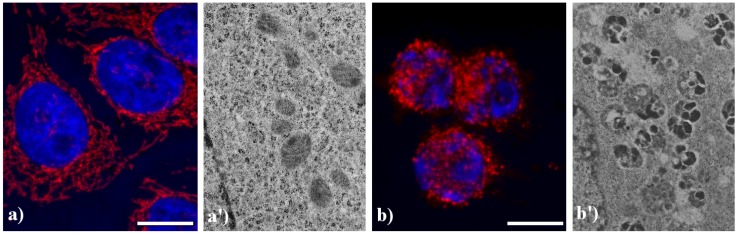
Morphological features of mitochondria. (**a**, **a'**) control HeLa cells; (**b**, **b'**) HeLa cells treated with etoposide (40 μM for 20 h). (**a**, **b**) confocal microscopy of mitochondria immunolabeled with a mouse monoclonal antibody recognizing the mitochondria-specific mtHSP70 (1:50 in PBS; Alexis, Vinci Biochemicals) and then with an Alexa 594-conjugated anti-mouse IgG antibody (1:200 in PBS, red fluorescence; Molecular Probes). DNA was counterstained with Hoechst 33258 (blue fluorescence). Scale bar: 20 μm. (**a'**, **b'**) electron micrographs.

Figure 5 shows the main modifications of mitochondria during apoptosis. In untreated HeLa cells (a), mitochondria are uniformly distributed throughout the cytoplasm showing the typical elongated shape; after the treatment with etoposide (b), mitochondria are fragmented, forming aggregates and more packed masses in the cytoplasm. The analysis at electron microscopy supports the evidence of damaged mitochondria during apoptosis: in control cells (a') internal cristae are still visible and conserved, while in treated samples (b') they result strongly compromised and mitochondria appear smaller, swollen and vacuolated.

The structural alterations occurring in mitochondria during apoptosis entail functional alterations, culminating in the release of mitochondrial proteins, including cytochrome c, Endonuclease G, the serine protease HtrA2, also known as Omi, Smac (Second Mitochondria-derived Activator of Caspases), DIABLO (Direct IAP-Binding protein with Low PI) and AIF (Apoptosis Inducing Factor) [25]. Accumulating evidence supports the notion that during apoptosis AIF is released from mitochondrial intermembrane space to the cytosol and moves to the nucleus, where it promotes chromatin condensation and high molecular weight DNA degradation [26]. The changes in AIF localization have been monitored by light and electron microscopy by different groups, including ours, often coupling its analysis to a careful evaluation of mitochondria morphology [4,26,27]. 

**Figure 6:**
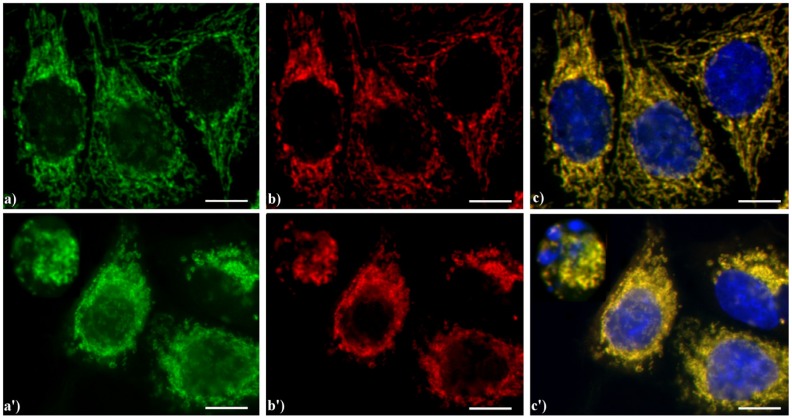
Double immunocytochemical detection of Apoptosis Inducing Factor (AIF) and mitochondria. (**a**–**c**) control HeLa cells; (**a'**–**c'**) cells treated with 10 μM etoposide for 20 h. Cells on coverslips were fixed and incubated with polyclonal anti-AIF antibody (**a**, **a'**) (dilution 1:50 in PBS; Cell Signaling Technology) and then with an Alexa 488-conjugated anti-rabbit antibody (1:200 in PBS, green fluorescence; Molecular Probes). Mitochondria (**b**, **b'**) were labeled with a mouse monoclonal antibody recognizing the mitochondria-specific mtHSP70 (1:50 in PBS; Alexis, Vinci Biochemicals) and then with an Alexa 594-conjugated anti-mouse IgG antibody (1:200 in PBS, red fluorescence; Molecular Probes). DNA was counterstained with Hoechst 33258 (blue fluorescence). The yellow staining in (**c**, **c’**) reveals that mitochondria (red fluorescence) and AIF (green fluorescence) colocalize. Scale bar: 20 μm.

As previously published, we observed that AIF translocates from mitochondria to the nucleus irrespectively of the applied apoptogenic stimuli, including etoposide, actinomycin D, carboxamides, cisPt, inhibitors of Na^+^/H^+^ exchangers and photosensitizers [4,23,24,27]. This event is widely reported to occur in early-mid phases of the apoptotic program. As an example, Figure 6 shows the yellow staining (c, c') supporting the evidence that in HeLa cells AIF (green fluorescence in a, a') colocalizes with the mitochondria-specific mtHSP70 (red fluorescence in b, b'), as expected for non-apoptotic cells (a–c), while the treatment with etoposide (a'–c') increases the expression of AIF both in the cytoplasm and nucleus in mid-apoptotic cells and only in the cytoplasm in late apoptosis, when nuclei are fragmented.

## 7. Conclusions

It is well known that apoptosis is associated to physiological programmed cell death, being fundamental during the embryonic development and in maintaining tissue homeostasis at the end of the histogenetic process [28,29]. In apoptosis, cell death is genetically regulated in response to a variety of stimuli and, *in vivo*, cells are rapidly removed by phagocytes without major perturbation of the tissue integrity.

Since the first reports published on apoptosis [30], the nucleus has especially been indicated as a marker organelle, and chromatin condensation and nuclear fragmentation have been described as the peculiar morphological signs of apoptotic cell death. In fact, during apoptosis the whole architecture of the cell nucleus is deeply affected, and in the last decade the ectopic reorganization of nuclear ribonucleoproteins (RNPs) into heterogeneous ectopic RNP-derived structures (HERDS) has also been described as a characteristic feature of apoptosis [31,32,33].

The findings summarized and discussed in this review suggest that also cytoplasmic organelles undertake largely stereotypic relocation and/or degradation during apoptosis, irrespective of the apoptogenic stimulus and cell type. In early apoptotic phases, microtubules and microfilaments are rearranged, with the formation of thick bundles especially at the cell periphery contemporary with the occurrence of changes to the plasma membrane, leading to the formation of surface blebbing and the release of cytoplasmic fragments. According to the literature, it is generally accepted that the restructuring of actin cytoskeleton is involved in the execution phase of apoptosis, including blebbing and karyorrhexis, and may influence the process of recognition and phagocytosis of apoptotic bodies [34]. On the other hand, microtubules are also important in the execution phase [35], when they form a cortical network under the plasmalemma, thus preserving cell morphology and plasma membrane integrity. The maintenance of this microtubule structure depends on high cellular ATP levels, so that microtubules undergo disorganization in late apoptosis, when mitochondria suffer extensive depolarization [36]. 

Using drugs that influence actin stabilization/depolymerization, it has been shown that stimulating alterations in the microfilaments status is sufficient to induce apoptosis. It is possible that variation in the endogenous balance between actin monomers and polymers may modulate the signaling to apoptosis, with alterations to actin providing the sensory mechanism [37]. Previous results by our group [3,38,39] demonstrate that different apoptogenic stimuli that do not directly affect the actin status are able to induce similar reorganization of the microfilament cytoskeleton, suggesting that these structural rearrangements may be viewed as an early morphological sign of apoptosis.

The same seems to be true in apoptotic cells for the ER and the GA, which break up and relocate during the early phases of apoptosis, and are released later inside apoptotic bodies. The accurate organization and function of the ER is essential for cell survival. Various events collectively termed *ER stress* (e.g., the inhibition of protein glycosylation, calcium depletion from the ER lumen, impairment of protein transport from the ER to the GA) can affect ER functions, inducing cellular damage and triggering apoptosis [40]. 

With its location in the center of the cell and its role in membrane trafficking, the GA is an ideal organelle to sense and integrate information about the cell functional state [41]; in addition, the continuous exchange by membrane fission/fusion between the ER and GA may allow integrating apoptotic signaling between these compartments during cell death induction and progress. Cleavage of ER and GA proteins by caspases certainly contributes to the disassembly of these organelles in apoptotic cells and is required for packaging of their fragments inside apoptotic blebs [42].

It is worth noting that the different organelles have reciprocal interactions with each other, both in viable and apoptotic cells. GA fragmentation was observed to occur in non-apoptotic cells upon microtubule depolymerization or when dynein (a microtubule motor protein) is inhibited [43]; similarly, in the apoptotic cytoplasm, microtubules are disassembled and dynein is inhibited by caspase cleavage [44]; this event could synergistically contribute to the observed dismantling of the GA.

The actin cytoskeleton and microtubules can also interact with mitochondria [45], which are considered as the central processing organelles in most apoptotic pathways. Apoptotic signals (either from outside or inside the cell) converge to mitochondria that can initiate the execution phase of apoptosis. The mitochondrial stress pathway entails the loss of transmembrane potential and the release into the cytoplasm of pro-apoptotic factors (among which, cytocrome c and AIF), thus contributing to the formation of the apoptosome [46]. These events may be followed by light microscopy using suitable cytochemical techniques such as staining living cells with JC1 (5,5',6,6'-tetrachloro-1,1',3,3'tetraethylbenzimidazolylcarbocyanine iodide) or immunolabeling mitochondrial pro-apoptotic factors, and may be morphologically detected by transmission electron microscopy. 

We may conclude that microscopy and cytochemistry still represent a suitable approach for identifying apoptotic cells through the detection of typical features in cytoplasmic organelles, starting in the early phases of the death process.
